# Study and Evaluation of a PCB-MEMS Liquid Microflow Sensor

**DOI:** 10.3390/s101008981

**Published:** 2010-10-08

**Authors:** Anastasios Petropoulos, Grigoris Kaltsas

**Affiliations:** 1 Institute of Microelectronics, NCSR-“Demokritos”, P.O. Box 60228, 15310, Aghia Paraskevi, Athens, Greece; E-Mail: greenewal@yahoo.com; 2 Department of Electronics, Technological and Educational Institute of Athens, 12210, Aegaleo, Athens, Greece

**Keywords:** microflow sensor, liquid flow measurement, PCB-MEMS

## Abstract

This paper presents the evaluation of a miniature liquid microflow sensor, directly integrated on a PCB. The sensor operation is based on the convective heat transfer principle. The heating and sensing elements are thin Pt resistors which are in direct electrical contact with the external copper tracks of the printed circuit board. Due to the low thermal conductivity of the substrate material, a high degree of thermal isolation is obtained which improves the operating characteristics of the device. The sensor is able to operate under both the hot-wire and the calorimetric principle. In order to fully exploit the temperature distribution in the flowing liquid, multiple sensing elements are positioned in various distances from the heater. A special housing was developed which allowed implementation of the sensor into tubes of various cross sectional areas. The sensor sensitivity and measurement range as a function of the sensing element distance were quantified. A minimum resolution of 3 μL/min and a measurement flow range up to 500 μL/min were achieved.

## 1. Introduction

Several Si-based thermal flow sensors are currently available for measuring gas and liquid flow rates [1–9]. These devices are typically mounted on a printed circuit board (PCB), whereby the connection of the sensor elements to the copper tracks is made via wire bonding. Due to the high thermal conductivity of the silicon substrate, various schemes are implemented in order to achieve the thermal isolation of the sensing elements, such as free standing structures, vacuum cavities or the formation of porous silicon. In this paper we present the evaluation of a miniature thermal liquid flow sensor fabricated by a technology which allows the device integration directly on printed circuit boards, as presented in [10]. Multiple advantages arise from the combination of PCB and MEMS techniques, such as the elimination of the need for wire bonding, the low production cost, the reduced amount of time required for the fabrication of the final device, as well as the relatively planar surface of the sensor which allows for minimally invasive flow measurements. A major advantage arising from the adoption of organic material as the device substrate is the very low heat dissipation that can be achieved, which provides highly effective thermal isolation without the need for additional fabrication stages.

Typically, thermal flow sensors contain a heating element which creates a local temperature increase and sensing elements that aim to measure the distortion in the temperature field as induced by the fluid flow. The two main principles of operation are the hot-wire and the calorimetric one. The hot-wire principle presents the simplest measuring method, as it requires a simple resistor which acts at the same time as both the heating and the temperature sensing element. In the calorimetric principle, the temperature difference between sensing elements located at a certain distance from the heater is monitored as a function of the flow rate. General advantages of the calorimetric principle are the possibility to detect the flow direction, the ability to detect lower flow rate values, the higher repeatability of the measurements and the faster response to flow rate changes.

In such case, the distances of the sensing elements to the heater is the dominant factor regarding the sensor performance; however a relatively limited amount of effort has been devoted in the study of the specific parameter. Volklein and Baltes in [11] presented a detailed analytical model whereby they quantify, among other quantities, the effect of the temperature sensing distance on the sensitivity of the device. They observed that the maximum sensitivity value is reached for high sensing element distance; however the device structure for which the specific model applies, incorporates a heat sink at a specific distance from the heater, with a corresponding boundary condition for the specific area to remain fixed at the ambient temperature. Such a condition is not true for most thermal flow sensor structures, including the one presented herein.

Nguyen and Dötzel used non-symmetrical distances of the sensing elements (from 200 μm to 660 μm), thus managing to increase the measurement range of their device to 150 mL/min, as compared to 10 mL/min which was the upper limit in the case of symmetrical positioning of the sensing elements [12]. Sabaté *et al.* used sensing elements at distances of 75, 150, 225 μm from the heater and found out that different sensing element combinations provided the optimum sensor signal at different flow rate regions. However they refered merely to symmetrical combinations as they didn’t examine the effect of the downstream and the upstream element distances independently. The measurement range of the device reaches up to 8 SLPM (gas flow) which cannot be considered extended. For the specific sensor the larger flow rates are detected by sensing element combinations with the smallest distance to the heater [13]. Kim *et al.* used sensing elements at varying distance (>1 mm) and concluded that small distance values increase the device sensitivity [14]. However according to the same authors, other groups (Mayer *et al.*) suggest that large distance values increase the device sensitivity. The specific contradiction is attributed to the order of magnitude of the sensing elements distance. In other words, for small distances (like Mayer *et al.* and Sabaté *et al.*) the increase in the sensing element distance increases the sensitivity of the device, while from a certain point on, the opposite holds. They support the existence of an optimum distance value, however they do not take under consideration the dependence of this distance value on the flow rate, or non symmetrical positions of the sensing elements with respect to the heater. Rasmussen *et al.* based on a numerical model, observe that the optimum distance of the sensing elements (being in symmetrical positions with respect to the heater) is a function of the fluid velocity. The trend is that for high velocities, the optimum sensing element distance is reduced (∼20–100 μm) [15].

Shin and Besser, making detailed calculations based also on a simulation model of their device, reach an optimum distance of 100 μm regarding the upstream sensing element, while regarding the downstream element, the optimum distance lies within a specified area (200–800 μm) depending on the flow rate. The specific analysis which is compatible with the operation of the PCB-MEMS flow sensor presented here, is not thoroughly verified by detailed experimental data [16].

Finally, van kuijK *et al.* and Durst *et al.*, reported on the effect of the sensing element distance to the heating source; however the devices they present are based on the time-of-flight operating principle and not the calorimentric one [17,18].

In general, most of the literature efforts to date tend to focus on isolated aspects of the overall effect that the positioning of the sensing elements has on the sensor operation. In this work we present a liquid flow sensor with a multiple resistor pattern, with the potential for appropriate selection of the resistor distance in order to obtain the optimum operating characteristics associated to a specific application. The systematic acquirement of operating data of the PCB-MEMS flow sensor is presented, which allows for a full characterization of the device. The study focuses on the effect that the independent parameters of the upstream and downstream element distance impose on the device sensitivity and measurement range.

## 2. Technology—Device Description

The presented thermal flow sensor is an alternative to Si-based sensors. The sensor elements are fabricated on top of a PCB, integrated directly to the copper tracks of the board. This way direct device communication to the macroworld is achieved without the aid of wire bonding, while the sensor control electronics can be implemented in the same substrate. A major advantage of having a polymer-based device is associated to the heat transfer rate from the heating element to the substrate. As both FR4 and SU-8, exhibit low thermal conductivity values (∼0.2 m·W/K), they provide an excellent thermal isolation platform for the sensor elements. Thus additional fabrication steps in order to achieve an isolation scheme are avoided, simplifying the overall process and minimizing the final cost and fabrication time. Furthermore, as no limitations are posed by the isolation scheme, large area structures can be realized. One more advantage of the proposed device is associated with its planar surface. Contrary to the common method of mounting the Si devices on a PCB, which inevitably introduces an obstacle to the fluid flow the height of which depends on the wafer thickness (>380 μm), the surface of the PCB-based device is relatively planar, allowing for minimally invasive flow measurements.

A detailed description of the fabrication technology used can be found in [10]. In this case, a standard PCB was used as a substrate on which copper tracks were first defined and a 15 μm thick SU8 layer was afterwards spin-coated and patterned. The heating and the sensing elements were 300 nm thick Pt resistors with lateral dimensions of 1.5 × 0.1 mm^2^. Direct electrical contact between the resistors and the copper tracks was formed through vias in the SU-8 planarization layer.

## 3. Operation Principle

Each resistor can act as both a heating element by utilizing the Joule effect, or as a temperature sensing element, since the resistor’s resistance is an explicit function of temperature according to:

(1) $$\frac{R(T)}{R(T_{o})}=1+\alpha \left(T-T_{o}\right)$$

with *R*(*T*) and *R*(*T_o_*) being the resistance values at a varying temperature *T* and a reference temperature *T_o_* respectively and α is the thermal coefficient of resistance (TCR). The average TCR of the fabricated resistors was 0.0024/°C. From (1) follows that the normalized resistance change is proportional to the temperature change imposed to a resistor:

(2) $$\frac{\Delta R}{R_{o}}=a\cdot \Delta T$$

where *ΔR = R*(*T_o_*) *− R*(*T*) and *ΔT = T_o_ − T*, respectively.

The monitoring of the normalized resistance change is necessary when measuring a large number of sensing elements, since it is a straightforward manifestation of the resistor temperature change, while at the same time it is independent of fabrication related features of each resistor such as its actual resistance value.

The liquids used in the conducted measurements were water and engine oil. As liquids exhibit high thermal conductivity values, the amount of thermal energy conducted away from a heating resistor is quite large. The temperature distribution within the fluid body in zero flow conditions is dictated by Fourier’s law of conduction:

(3) $$q_{c}=-k\nabla T$$

where *q_c_* is the conductive heat flux and *k* is the thermal conductivity of the liquid under test. For a constant heat flux the temperature gradient is low, signifying a non-localized temperature distribution within the liquid. The flow induced temperature change can take place in an area of a few centimeters, which is a rather large value concerning microdevice dimensions.

The sensor is able to operate under both the hot-wire and the calorimetric principles. The hot-wire operating principle involves the monitoring of the cooling effect on a single heating element and is relatively simple to realize. However operation under the calorimetric principle, whereby the temperature difference of two sensing elements situated in either side of the heater is monitored as a function of the flow rate, provides data of higher accuracy and reproducibility. A major advantage in this case is that the effect of heat conduction from or to the ambient is essentially cancelled out when subtracting the signal of the two sensing elements. Additionally, potential temperature fluctuations of the heating element pose a minimal effect on the final measurement. Obviously, for this to remain true the sensing elements have to be monitored at the same time. Therefore, since in our case a full distribution of the temperature field on the upper sensor surface was required, this could only be achieved by the simultaneous measurement of the involved sensing elements.

**Figure 1.**
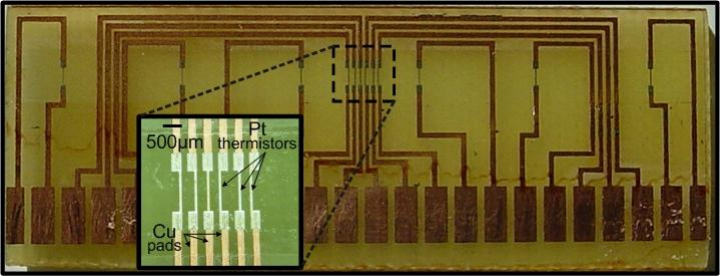
A photograph of the fabricated sensor. At the inset, an enlarged image of the dense part of the multiple resistor pattern is shown.

In order to achieve optimum operation in the calorimetric operating principle, the resistor pattern of the liquid flow sensor is designed in a way that the resistors are spread over a wide area so as to fully exploit the occurring flow induced temperature variations. As shown in Figure 1 the resistor pattern consists of multiple elements, designed in such a way that large distance values between the sensing elements and the heater are allowed. An area where the resistors are positioned in a dense manner is also included, while the maximum distance of the two outer resistors is 52 mm.

## 4. Measurement Setup

**Figure 2.**
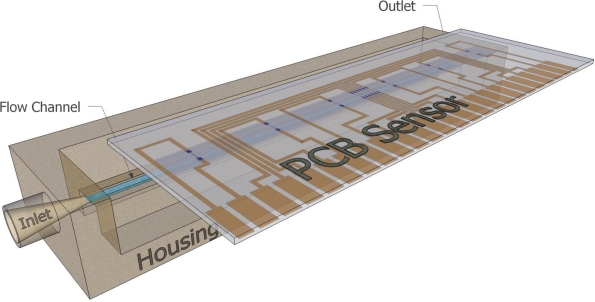
A schematic of the implemented housing. The sensor is adjusted on a special packaging with the resistors’ side facing the defined flow channel. Sealing is assured by a specific O-ring scheme (not shown in the picture).

The sensor was wall-mounted on a specially designed package which features a well defined flow channel, as well as a liquid inlet and outlet (Figure 2). The length of the flow channel was 6 cm, while its cross sectional area was 1.2 mm^2^. The packaging was specially constructed so as to accommodate the sensor board, with the sensing elements aligned to the flow channel axis. Sealing is maintained by a special type of O-ring adjusted next to the edges of the flow channel.

**Figure 3.**
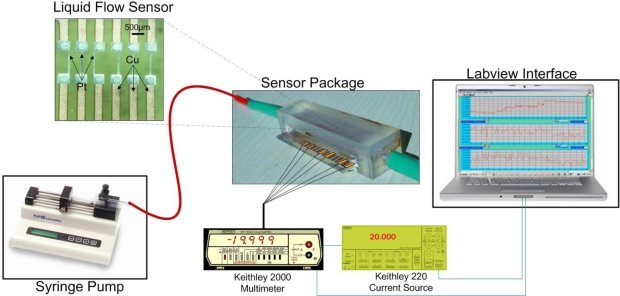
A schematic of the measurement setup used. A syringe pump provides the reference flow to the sensor package. A current source is used to provide the heater operating current, while the resistance variation of the resistors is recorded by a multimeter equipped with a 10-channel scan card. The obtained data are monitored through a PC.

The reference flow of liquid was provided by a WPI syringe pump in the region of 0–500 μL/min. Regarding the electronic equipment used, the heater was supplied by electrical current generated by a Keithley 220 current source, while the heater voltage was monitored by a Keithley 2000 Multimeter. The resistance values of the upstream and the downstream sensing elements in the calorimetric operating principle were measured by a second Keithley 2000 Multimeter equipped with a 10-Channel Scanner Card. The specific instrument alongside a specially designed Labview® interface enables the simultaneous recording of the resistance values of a maximum of ten sensing elements. All instruments were connected to a PC via a GPIB interface. A schematic of the measurement setup is shown in Figure 3.

The sensor can operate in either the CI mode, whereby the heater is supplied with a current of constant value, or in the CT mode, whereby the heater resistance and therefore the heater temperature is kept constant, regardless the fluid flow. Generally the CI mode is easier to implement, however the measurements conducted in the CT mode are more reliable and provide better resolution.

In order to obtain the CT operating mode an appropriate algorithm was developed in the Labview® environment. The software records the resistance value of the heater *R_h_* in regular time intervals and compares it to the nominal resistance value *R_c_* to which the heater resistance is meant to be stabilized. If │*R_h_ − R_c_*│ is greater than the minimum allowable resistance change *R_m_*, then the algorithm redefines the new current value so as for│*R_h_ − R_c_*│ to become less than *R_m_*. It is apparent that the lower the *R_m_* the more accurate the stabilization method is. Its value is mainly determined by the smallest controllable current variations in the output of the power source. The resolution of the current source at the operating region of interest (50–100 mA) is 5 × 10^−5^ A which leads to a value for *R_m_* of 5 × 10^−3^ Ω and an overall temperature stabilization error of 0.2 °C.

## 5. Results and Discussion

### 5.1. Introduction

The in-depth characterization of the sensor operation which is performed in this section is a rather demanding task, mainly due to the complexity arising from the multiple signals acquired from the resistor array. Initially, in a quantitative approach the sensor operation under the hot-wire principle is compared to operation under the calorimetric principle. Subsequently, an elaboration on the multiple resistor array is performed. The simultaneous monitoring of numerous resistors produces data of relatively large size. The recording and manipulation of the acquired data requires thorough processing in order to define the significant parameters and extract the optimum sensor performance.

The most important characteristics associated to the sensor operation are the exhibited measurement range, sensitivity and resolution. The effect of the distance of the upstream and the downstream sensing elements on these parameters will be analyzed. The test liquids used in the experiments were water and engine oils of varying viscosity.

### 5.2. Qualitative Comparison of Hot-Wire and Calorimetric Operation

In this section the sensor operation under the hot-wire and the calorimetric principle is compared. The general case regarding thermal flow sensors is that operation in the calorimetric principle provides data of increased resolution and lower minimum detectable flow rate. Contrary to that, in the hot-wire principle an extended measurement range is provided, as the signal reaches saturation at higher flow rate values.

**Figure 4.**
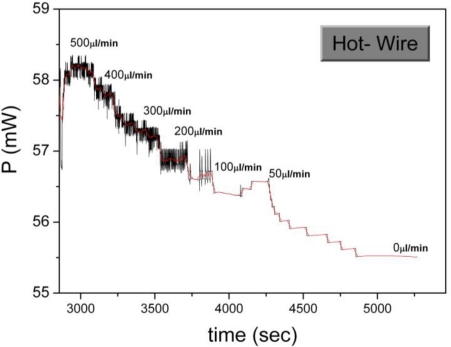
The sensor operation under the hot-wire principle (CT mode). The power generated at the heater for denoted flow rate values is shown. The black line represents the real time sensor signal, while the red line is derived by averaging of the results.

In the specific case the sensor operation under both operating principles was evaluated in the CT mode, so as to obtain measurements of maximum quality. In Figure 4, the sensor operation in the hot-wire principle is shown. The measured quantity is the power generated at the heater *P_i_* for the resistance stabilization and for varying flow rate. As it can be observed, for increasing flow rate, the generated power also increases. However, the obtained data are of poor quality, with a low signal to noise ratio and without a clear correlation of the sensor signal to the flow rate. Furthermore, a very low sensitivity is observed at the low flow rate region.

**Figure 5.**
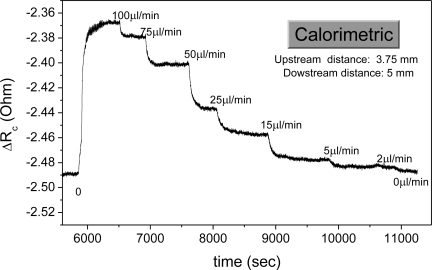
The sensor operation under the calorimetric principle, for *D^down^* = 5 mm and *D^up^* = 3.75 mm. The real time signal is the change in the resistance value of the sensing elements at the denoted flow rate values.

Concerning the calorimetric operating principle, we define the quantities *R^down^* as the resistance value of the downstream sensing element, *R^up^* as the resistance value of the upstream sensing element, while the corresponding distances of the sensing elements to the heater are termed *D^down^* and *D^up^*. In an initial approach, the sensor signal can be expressed as the change in the resistance of the downstream and the upstream sensing elements, defined as *ΔR_c_ = R^down^ − R^up^*. Figure 5 shows the real time sensor operation in the calorimetric operating principle.

It is observed that a clear correlation between the sensor response and the flow rate holds throughout the measurable flow rate region with high resolution in the low flow rate regime.

### 5.3. The Multiple Resistor Array

The results of the calorimetric operation as presented in the previous section refer to a single combination of *D^down^* and *D^up^*. The major advantage of the specific sensor however lies on the ability to implement numerous sensing element configurations, extracted from the multiple resistor array. However, the simultaneous monitoring of ten resistors produces data of relatively big size as a large number of sensing element combinations can take place. The quantity which can depict the actual change in the temperature field in a rather global approach is the difference in the normalized resistance change, defined as:

(4) $$\Delta R_{a}=\frac{\Delta R^{\mathrm{down}}}{R_{o}^{\mathrm{down}}}-\frac{\Delta R^{\mathrm{up}}}{R_{o}^{\mathrm{up}}}$$

with $\Delta R^{\mathrm{down}}=R^{\mathrm{down}}-R_{o}^{\mathrm{down}}$, $\Delta R^{\mathrm{up}}=R^{\mathrm{up}}-R_{o}^{\mathrm{up}}$, while $R_{o}^{\mathrm{down}}$ and $R_{o}^{\mathrm{up}}$ being the resistance values of the downstream and the upstream elements at zero flow respectively. Based on equation (2), the latter becomes:

(5) $$\Delta R_{a}=a\cdot \Delta T^{\mathrm{down}}-a\cdot \Delta T^{\mathrm{up}}=a\cdot \Delta T_{c}$$

where *ΔT^down^* and *ΔT^up^* are the change in temperature in the downstream and the upstream sensing element respectively, while *ΔT_c_ = ΔT^down^ − ΔT^up^* is the temperature difference of the sensing elements. Therefore by monitoring *ΔR_α_* one knows the actual temperature difference between two resistors positioned in specified distances from the heater. This comes as a necessity due to the fact that the actual resistance values of the fabricated resistors may vary, which inevitably affects *ΔR_c_*. *ΔR_α_* however is independent of the actual resistor resistance values, since it depicts merely the temperature differences between specified points. This feature allows for a study of the phenomenon in a global approach, as the obtained results are not related to fabrication issues, while data obtained from different measurements and potentially different sensors with alternate resistor patterns can be comparable.

**Figure 6.**
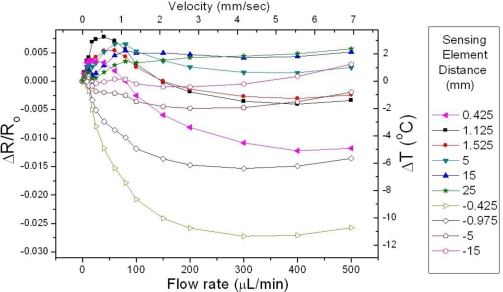
Normalized resistance change of the sensing elements situated in various distances from the heater as a function of the flow rate. The liquid used was water, while the tube crossectional area was 1.2 mm^2^.

In Figure 6, the normalized resistance change *ΔR/R_o_* of ten sensing elements as a function of the flow rate is shown, for heater operation in the CI mode and water as the test liquid. The distance of the downstream sensing elements *D^down^* to the heater is denoted with a positive sign, while the distance of the upstream sensing elements *D^up^* is indicated with the negative values. As a general remark, it is observed that the signal of the upstream sensing elements is a monotonically decreasing function of the flow rate throughout the entire measurement range. Contrary to that the signal of the downstream sensing elements increases up to a saturation point, the magnitude and the location of which depend on *D^down^*.

### 5.4. Data Manipulation

The curves shown in Figure 6 correspond to the raw form of the data acquired in a single measurement. The manipulation of the provided information in order to extract the optimum sensor performance is a non-trivial issue. Evidently, there is a very a large number of combinations which can form the calorimetric sensor signal *ΔR_α_*. The method chosen in order to provide the means to systematically study the sensor behavior is to construct a sequence of “facets”; in each facet one specific upstream element is chosen to be maintained as a reference, while the sensor response as a function of the flow rate for the remaining sensing elements (each one termed “local element”) is depicted. The distance of each local element to the heater is denoted as *D^loc^*, which is assigned positive values for the downstream elements and negative values for the upstream elements.

The explicit form of the sensor signal in each facet is defined as:

(6) $$\Delta R_{\alpha }^{i}=\frac{\Delta R^{i}}{R_{o}^{i}}-\frac{\Delta R^{\mathrm{up}}}{R_{o}^{\mathrm{up}}}$$

with $\frac{\Delta R^{i}}{R_{o}^{i}}$ being the normalized resistance change of each of the local elements. Given that in each facet there is one reference sensing element combined with all the remaining local elements, it can be deduced that the signal of resistors lying on the same side with the reference upstream sensing element with respect to the heater is also incorporated. Despite the fact that such a combination of sensing elements is uncommon, it yields interesting results as seen in the following graphs.

By successively performing this task, facets corresponding to all potential *D^up^* values are constructed. The assembly of the constructed facets, leads to the creation of a “frame” which essentially includes all the information extracted by a measurement, organized in a structured manner, thus allowing for the characterization of the sensor behavior regarding a specific fluid.

**Figure 7.**
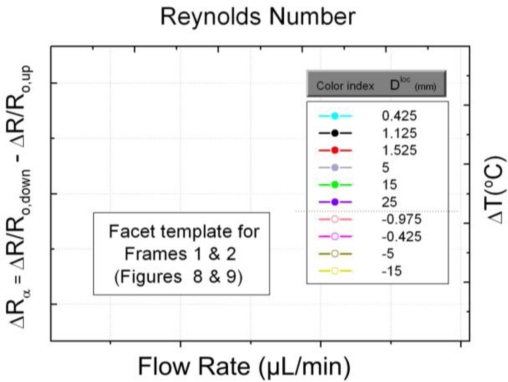
Description of the template which applies for all facets used in the frames. On the left y-axis *ΔR_α_* is shown, while the corresponding *ΔT* is shown on the right y-axis. The bottom and the top x-axis show the flow rate and the Reynolds number respectively. In the legend, the correspondence between the line color and the distance of each sensing element to the heater is given.

In the specific case, each frame consists of four facets as is the number of the upstream sensing elements used. The information contained in each facet is depicted in the standard template form outlined in Figure 7. The left y-axis shows *ΔR_α_* as defined in (6), while the right y-axis shows the corresponding temperature difference as defined in (5). The bottom x-axis shows the flow rate values (in μL/min), while the top x-axis shows the corresponding Reynolds numbers as defined by [19]:

(7) $$\text{Re}=\frac{\rho \mathrm{uD}}{\mu }$$

where *ρ* is the fluid density, *u* is the mean velocity of the fluid, *D* is the tube diameter and *μ* is the dynamic viscosity of the fluid. The use of Reynolds number provides a more global way of quantifying the fluid flow. Finally, the legend shows the association of the color index used in the facets with the distance of the local sensing elements.

**Figure 8.**
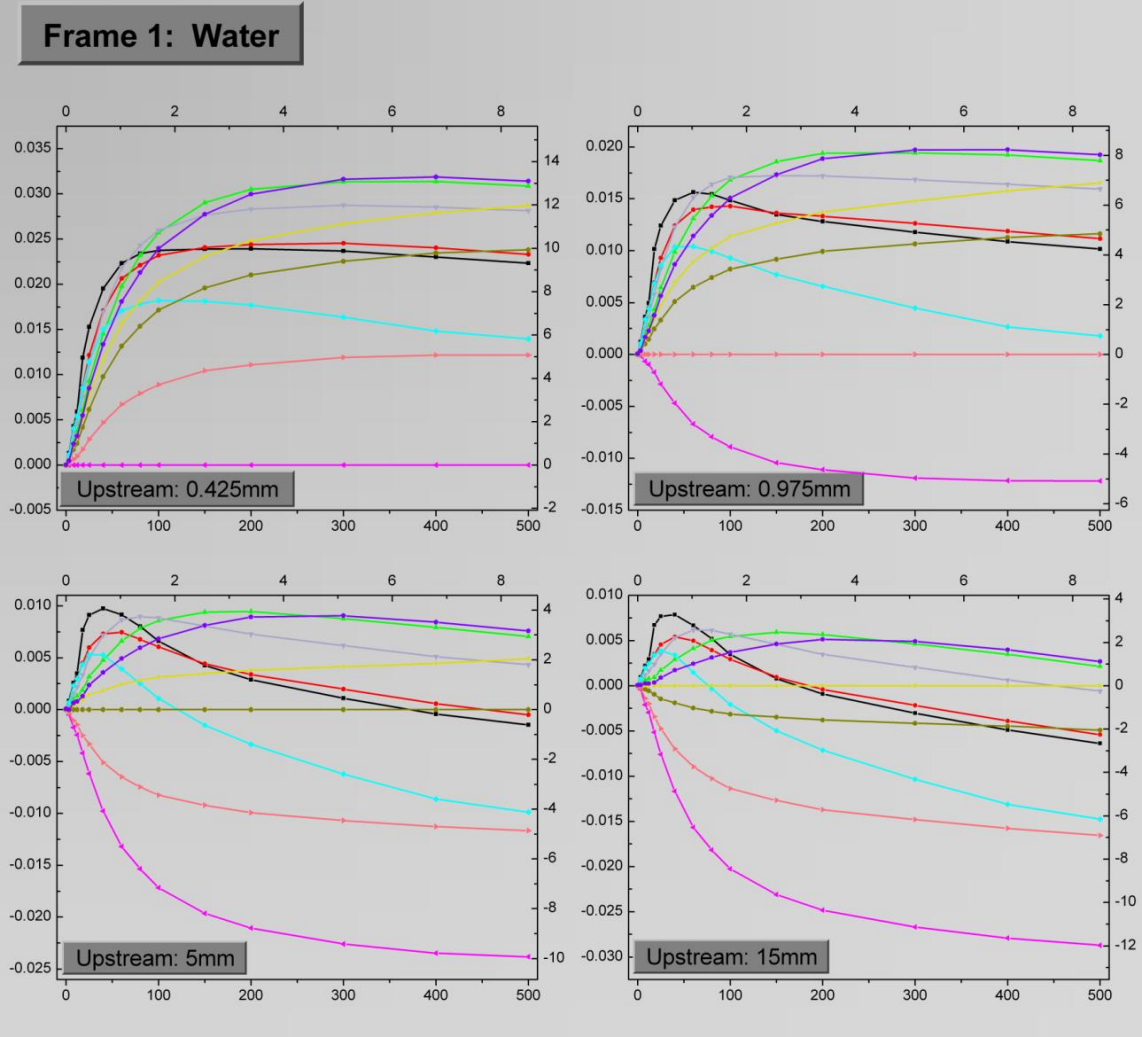
Frame 1 which describes sensor operation for water is shown. Each facet is associated to a different *D^up^* value, while each curve corresponds to a certain *D^loc^*. The axes quantities as well as the correspondence between the line colors and *D^loc^* are explained in Figure 7.

**Figure 9.**
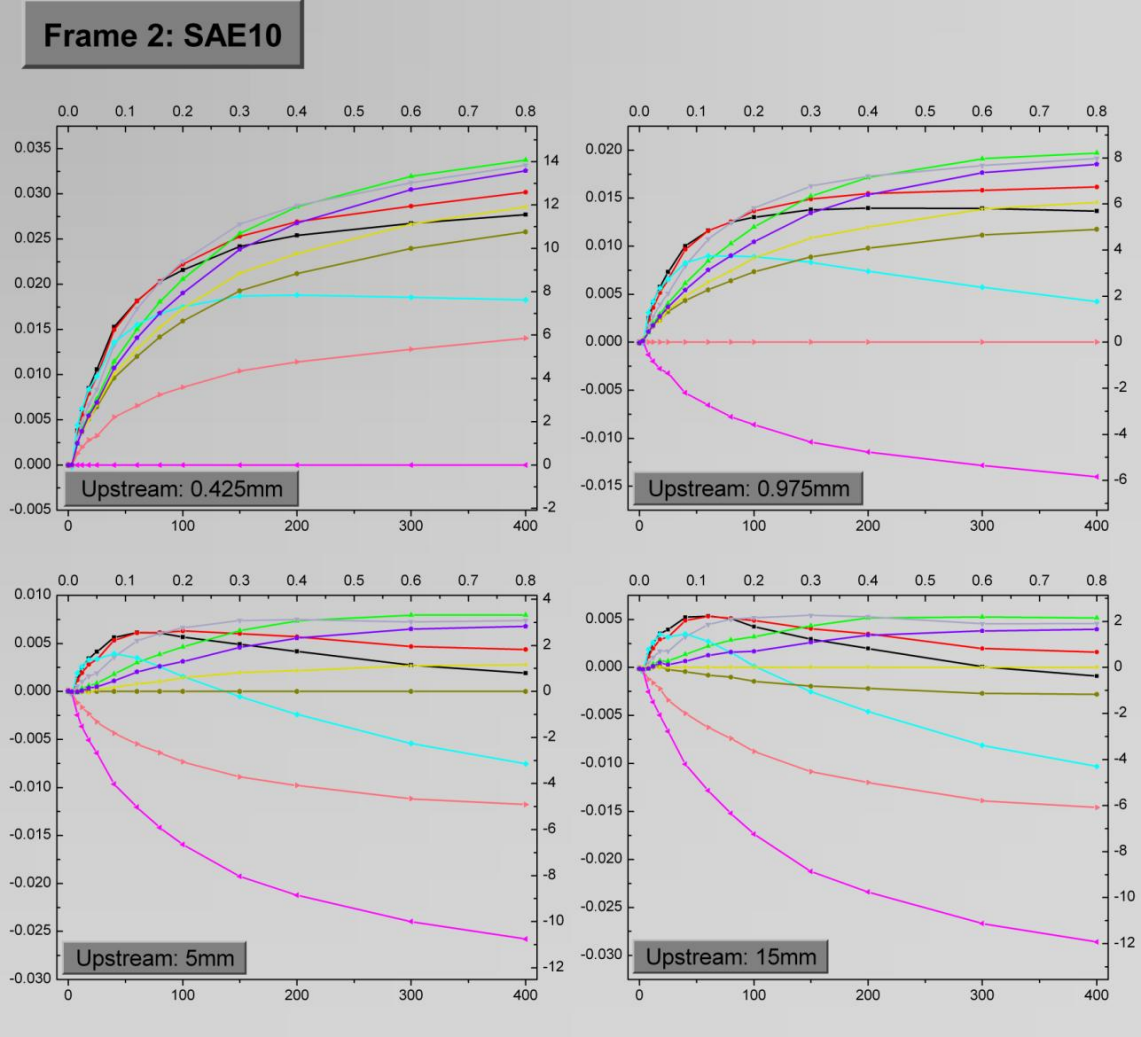
Frame 2 which describes sensor operation for the engine oil with viscosity SAE10. The correspondence between *D^loc^* and the line colors is maintained.

The four-facet frames corresponding to water and to the engine oil with viscosity equal to SAE10 as the test fluids are depicted in Figures 8 and 9, respectively. The *D^up^* values for the facets are 0.425 mm, 0.975 mm, 5 mm and 15 mm respectively. Each facet shows the variation of *ΔR_α_* for the ten sensing elements as a function of the flow rate. The general trend is that *ΔR_α_* is initially an increasing function of the flow rate, up to a saturation point, the location of which depends on the sensing element combination. A straight line at each facet which corresponds to *ΔR_α_* = 0 occurs when the upstream sensing element is subtracted from itself, as stems from (6). Obviously these lines do not hold any information, but they are maintained in the graphs for reference purposes.

As evident, there is not a specific sensing element combination which can provide optimum results in terms of sensitivity and measurement range. In general, large *D^loc^* result to extended measurement range, while high sensitivities, especially in the low flow rate region are extracted from low *D^loc^* values. The precise effect of *D^loc^* on these quantities will be discussed in the following paragraphs.

The effect of *D^up^* on the sensor signal is more straightforward. By observing the sensor signal it is deduced that the optimum results come for *D^up^* = 0.425 mm, which is the lowest value. For relatively large *D^up^* the highest sensor signal in absolute values, is the one derived by subtracting the signal of sensing elements both lying on the downstream side. Finally, when comparing the results in the two frames, a rather general remark is that for identical sensing element combinations water exhibits higher sensitivity for low flow rates, while in the oil case a wider measurement range is observed.

### 5.5. Measurement Range

A brief mention regarding the effect of the sensing element distance on the measurement range was given in the previous paragraph. In order to provide a more detailed insight on this effect, in a first approach we define the measurement range of the device as the flow rate region up to which *ΔR_α_* is an increasing function of the flow rate. In order to provide a more qualitative study on this effect, we have not included those combinations that are composed of two downstream sensing elements, which provide enlarged measurement range values. In other words, the combinations taken under consideration are composed of one upstream and one downstream sensing element.

**Figure 10.**
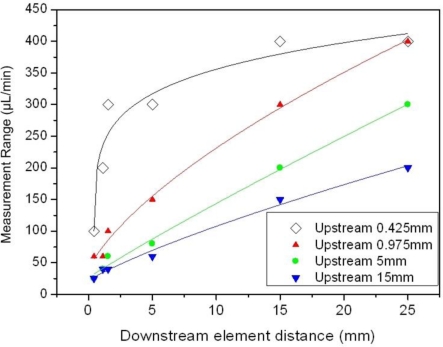
The measurement range as a function of the downstream element distance. The data are extracted form Frame 1 (water as the test fluid). Each facet is represented by a different color as indicated in the legend.

The signal saturation point, which defines the corresponding measurement range, as a function of *D^down^* for the various *D^up^* values that correspond to the facets of Frame 1 are given in Figure 10. Each point in the graph corresponds to the saturation point for a certain configuration of *D^down^* and *D^up^*, while each facet is represented by a certain color. It is apparent that the measurement range is an increasing function of *D^down^* for distances up to 25 mm. This is considered a very large value regarding typical microsensor dimensions.

Regarding the effect of the upstream sensing element distance, it is seen that for a certain *D^down^* the measurement range increases as *D^up^* decreases. This holds up to *D^down^* = 25 mm, whereby the measurement range for *D^up^* = 0.425 mm and *D^up^* = 0.975 mm is identical. Generally, for *D^up^* = 0.425 mm the measurement range is essentially stabilized at 400 μL/min for *D^down^* > 15 mm, while for larger *D^up^* values the measurement range remains an increasing function of *D^down^*. The trend of the curves suggests that although there is an optimum *D^up^* for the adopted *D^down^* values herein (up to 25 mm), an extension in the measurement range can be achieved for *D^down^* larger than 25 mm and *D^up^* = 0.975 mm or more.

Overall, the presented results suggest that an increase in the measurement range is obtained by increasing *D^down^*, while *D^up^* = 0.425 mm is the optimum distance since in that case the largest measurement range is presented for a specific *D^down^* value. For a more precise study on the effect of the specific parameters on the measurement range one would need a resistor pattern with more than ten elements positioned within a specified space, as well as smaller transition steps of the flow rate during the measurement procedure. In such case the curves of Figure 10 would be smoother, presenting a more accurate depiction of the actual phenomenon, *i.e.*, the temperature distribution on the sensor surface. Although this is an intriguing prospect which can be considered helpful in terms of a theoretical approach, it is however beyond the scope of the present work which aims for a characterization of the sensor operation.

### 5.6. Sensitivity and Resolution

A crucial parameter regarding the sensor operation is the exhibited sensitivity throughout the entire flow region. In general terms the device sensitivity *S_c_* of a flow sensor expresses the rate of change of the sensor signal with respect to the flow rate *Q* at a specific flow rate value *Q_0_* and can be defined as:

(8) $$S_{c}(Q_{0})=\frac{d(\Delta R_{\alpha })}{dQ}{|}_{Q=Q_{0}}$$

However, since during the device evaluation the changes in the flow rate are performed discretely, a compatible way to represent sensitivity is required. In this context we denote the constant flow regions *Q_k_*, with the index *k* = 1,2,3… being a number assigned to each constant flow rate region in a sequential manner. We define the change of the flow rate occurring between two neighboring constant flow regions as *ΔQ_k_ = Q_k+1_ − Q_k_*, and also define the variable *q_k_* as:

(9) $$q_{k}=Q_{k}+\frac{\Delta Q_{k}}{2}$$

which is essentially used to express the midpoint of *ΔQ_k_* .

In this context the average sensor sensitivity is defined as:

(10) $$S_{d}(q_{k_{0}})=\frac{\Delta (\Delta R_{\alpha })}{\Delta Q_{k}}{|}_{k=k_{0}}=\frac{\Delta (\Delta R_{\alpha })}{2(q_{k}-Q_{k})}{|}_{k=k_{0}}$$

where *Δ*(*ΔR_α_*)= *ΔR_α,k_* − *ΔR_α,k+1_*, with *ΔR_α,k_* and *ΔR_α,k+1_* the sensor signal at the corresponding flow rate values *Q_k_* and *Q_k+1_*. At the limit where *ΔQ_k_* → 0, *S_d_* coincides to *S_c_*. The units regarding both sensitivity quantities are (μL/min)^−1^.

As it can be extracted from Figures 8 and 9, the sensor signal is not a linear function of the flow rate, therefore *S_d_* is not constant. Due to the large number of sensing element combinations, an equal number of individual “pair sensitivities” $S_{a}^{i}$ exists regarding a single *q* value of the flow rate. The optimum sensor sensitivity $S_{d}^{m}$ at a specific *q* equals to the maximum of the $S_{a}^{i}$ as calculated throughout each frame.

**Figure 11.**
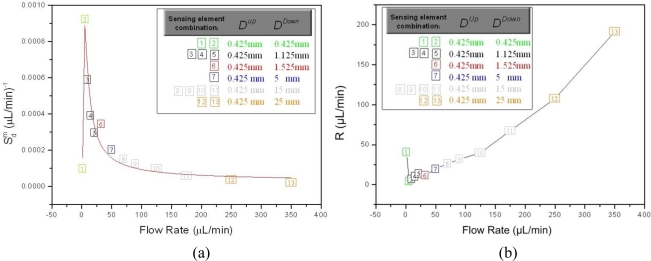
(a) The sensor sensitivity $S_{d}^{m}$ as a function of the flow rate, as indicated in Table 1. The color of each point denotes the corresponding sensing element combination which exhibits the specific sensitivity value, while the associated *k*-value is also depicted. The color of each point corresponds to a certain sensing element combination, as indicated in the legend. (b) The resolution R of the sensor as a function of the flow rate.

By using the proposed formalization, it is possible to define the sensor resolution *R*, which denotes the minimum change in the flow rate which can be detected by the device. The main factor that limits the sensor resolution is the noise level associated to the implemented electronics. By defining *n* as the minimum value of *ΔR_α_* which is detectable by the employed system, it is easily derived that the sensor resolution is inversely proportional to the overall sensor sensitivity as in:

(11) $$R(q)=\frac{n}{S_{d}^{m}(q)}$$

where the resolution units are μL/min. In Table 1, the results of the calculated values regarding the sensor sensitivity $S_{d}^{m}$ and the associated resolution *R* at each *q* are summarized, while the corresponding sensing element combination for each sensor sensitivity value, is also denoted. The data correspond to Frame 2 (engine oil SAE 10). A graphical representation of the obtained results is shown in Figure 11a regarding the sensitivity and in Figure 11b regarding resolution.

The x-axis of each point in the graphs of Figure 11 represent *q,* while the y-axis corresponds to $S_{d}^{m}$ (Figure 11a) and *R* (Figure 11b). The color of each point is assigned to a certain sensing element combination as defined in the graph legend, while the incremental numerical value of each point depicts its corresponding *k* index as defined in Table 1.

As observed in the derived results, the sensing element with *D^up^* = 0.425 mm is incorporated in all the sensing elements combinations that comprise the maximum calculated sensitivity, throughout the entire measurement range. This clearly implies that in terms of sensitivity the specific upstream distance is the optimum choice concerning the distance values used in this experiment. Regarding the curve form, it is seen that sensitivity reaches to a maximum at *q* = 5.5 μL/min and from that point on it remains a decreasing function of the flow rate. The initial increase from *q* = 1.5 μL/min to *q* = 5.5 μL/min is a direct consequence of the S-shaped response of the device to the flow change, which is a typical sensor characteristic [20]. From a qualitative point of view, the most significant observation is that for larger values of *q*, *D^down^* also increases. This result is compatible to the discussion in the previous paragraph, *i.e.*, that large *D^down^* values allow for an increased measurement range; however, the provided *S_n_* for *q* larger than 50 μL/min is reduced significantly. An analogous situation applies regarding the variation of resolution, with its minimum value being 4.34 μL/min at *q* = 5.5 μL/min, while it rises up to 191.39 μL/min for *q* = 350 μL/min.

## 6. The Effect of the Crossection Area on the Sensor Operation

**Figure 12.**
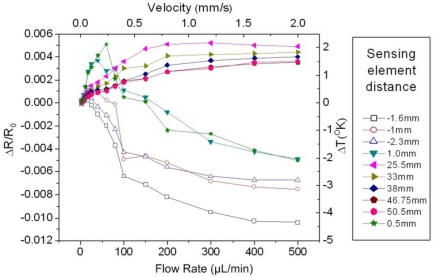
The normalized resistance change of the sensing elements as a function of the flow rate, for water as the test liquid. The distance of each sensing element to the heater is depicted, with the negative values corresponding to the upstream sensing elements. The tube crossectional area is 4.2 mm^2^.

The analysis of the results conducted so far refers to a tube of certain crossectional area (1.2 mm^2^). In order to study the effect of the specific parameter we used a configuration with a tube of similar length (6 cm), but a with a larger crossectional area of 4.2 mm^2^. The resulting data are shown in Figure 12, which is an analogue of Figure 6 for the specified cross sectional area value. The dependence of the normalized resistance change *ΔR/R_o_* of ten sensing elements as a function of the flow rate is presented.

The positioning of the sensing elements in space is different in this case, as can be noticed from the distance values of *D^up^* and *D^loc^* which are noted at the legend of Figure 12. The detailed observation of the specific graph leads to important conclusions which are verified by the entire set of measurements conducted for the 4.2 mm^2^ tube:

- Sensitivity: As clearly observed in the graph, the changes of *ΔR/R_o_* with the flow rate are smaller compared to the corresponding ones of Figure 6. This can be interpreted as a generally reduced sensitivity for the increased crossection area tube.
- Measurement range: The measurement range remains practically unaltered, as observed in the series of experiments conducted in both cases. This applies for both the minimum detectable flow rate, as well as for the upper measurement limit.
- Optimum *D^up^* value: From the analysis of the results in the case of the small crossectional area tube which has been carried out in the previous paragraphs, it is seen that the minimum distance value of the upstream sensing element *D^up^* = 0.425 mm is the optimum, concerning both the measurement range and the sensitivity of the device. Due to this, one could be led to the conclusion that further reduction of *D^up^* could in principle enhance the sensor operation, an hypothesis that might hold true up to a point.

For the measurement indicated in Figure 12, three upstream sensing elements have been used, with *D^up^* values of 1 mm, 1.6 mm and 2.3 mm. In general terms, the value which provides the optimum signal is *D^up^* = 1.6 mm, and not the minimum one. This leads to the conclusion that in the general case the optimum *D^up^* value is a function of the crossectional area. These finding is highly useful concerning the understanding of the sensor operation, as it implies that an optimum upstream distance value exists, which is a function of the crossectional area. Consequently, it can be extracted that the optimum *D^up^* in the 4.2 mm^2^ crossection case can be defined as 1 < *D^up^* < 2.3 mm, while in the 1.2 mm^2^ case the corresponding range is *D^up^* < 0.975 mm.

## 7. Comments

Typically, sensors that measure liquid flow rates in the μL/min region, based on the thermal principle, employ a microchannel of significantly smaller cross section dimensions compared to the one implemented in the specific case [21,22]. The ability to detect temperature differences induced from such low flow velocities is attributed to the high level of thermal isolation achieved by the polymer materials that comprise the device. This feature, along with the utilization of the multiple resistor pattern, allows for a measurement range that is well over two orders of magnitude, which is an outstanding value for operation under the calorimetric principle, compared to thermal flow sensors presented in the literature [2].

The presented results regarding the device operation refer to water and to engine oil with viscosity SAE10 as test liquids. No explicit attempt to quantify the dependence of the operational parameters on the thermal properties of the test liquid in terms of the exhibited thermal conductivity *k* and heat capacity *C_p_* was made; however the invariance of the sensor performance on the mechanical properties of the fluids involved was verified experimentally. This was achieved by performing analogous measurements with engine oils with SAE20 and SAE40 viscosity values as test fluids, which differ from SAE10 in terms of viscosity. An identical sensor response was observed in all three cases.

Furthermore, there is no influence of the ambient temperature on the sensor performance. Specifically, in the calorimetric principle, a potential change in the external temperature affects equally all sensing elements, and since the final sensor signal is comprised of the subtraction of two element signals, the overall temperature effect is cancelled out. In the hot-wire principle, a very simple method in order to compensate for potential changes of the ambient temperature, is to incorporate a sensing element away from the heater’s thermal field to serve as a temperature reference. By subtracting the sensing element signal from that of the heater, the ambient temperature effect is cancelled out. However, due to the adequate temperature control during the conduction of the measurements, such a scheme was not implemented.

Beyond the conducted evaluation of the sensor performance, the employed multiple resistor array has provided an insight on the distribution of temperature field on the device surface under liquid flow. It was experimentally verified that the flow defined temperature field generated by a heating resistor on a thermally isolated surface can produce exploitable temperature differences at distances far larger than the typical dimensions of micromachined sensors. The obtained data can provide a useful experimental reference in an analytical description of the temperature field under fluid flow.

As previously stated, no specific combination of the sensing elements distance provides optimum results throughout the entire measurement range. In order to fully exploit the sensor potential in terms of measurement range and sensitivity, one needs to be able to tune the device in such a way that the appropriate sensing element combination is selected for a specified application. This task can be achieved by implementing a microcontroller which would appoint the active sensing elements for a specific measurement based on an initial flow rate estimation as derived by the sensing element combination which provides the most extended measurement range. The development of the appropriate interface electronics that are meant to carry out this process is in progress.

## 8. Conclusions

The detailed evaluation of a polymer-based liquid flow sensor is presented in this paper. The device is based on a technology that allows the development of thermal sensors on printed circuit boards, whereby direct device communication to the outside world is maintained without the use of wire bonding. The sensor presents highly satisfactory operational characteristics which are mainly attributed to the high level of thermal isolation of the heating and sensing elements due to the low thermal conductivity exhibited by the polymer materials that comprise the device. The sensor operation was tested in both the hot wire and in the calorimetric principle. The key attribute of the presented sensor is the multiple resistor pattern employed, which features sensing elements in largely varying distances from the heater so as to be able to fully exploit the temperature changes occurring in a large distance from the heating element. By categorizing and processing the data provided by the ten resistors, the effect of both the upstream and the downstream element distance on the device operation in terms of measurement range, sensitivity and resolution was quantified. The minimum detectable flow rate was 2 μL/min which corresponds to a Reynolds number of 0.03, while the measurement range extends over 400 μL/min. By the proper selection of the active sensing elements, the optimum device operation for a specified application can be obtained.

## Figures and Tables

**Table 1.** The information regarding the device sensitivity, resolution as well as the corresponding sensing element combination as extracted by processing the data of Frame 2.

***k***	***Q_k_* (μL/min)**	***ΔQ_k_* (μL/min)**	***q* (μL/min)**	$S_{d}^{m}$**(10^−5^ · (μL/min) ^−1^)**	***R* (μL/min)**	***Sensing Elements***	
						***D^up^* (mm)**	***D^down^* (mm)**

1	0	3	1.5	9.87	40.52	0.425	0.425
2	3	5	5.5	92.20	4.34	0.425	0.425
3	8	4	10	58.75	6.81	0.425	1.125
4	12	6	15	39.17	10.21	0.425	1.125
5	18	7	21.5	29.57	13.53	0.425	1.125
6	25	15	32.5	34.33	11.65	0.425	1.525
7	40	20	50	20.35	19.66	0.425	5
8	60	20	70	15.15	26.40	0.425	15
9	80	20	90	12.55	31.87	0.425	15
10	100	50	125	10.06	39.76	0.425	15
11	150	50	175	5.92	67.57	0.425	15
12	200	100	250	3.71	107.82	0.425	25
13	300	100	350	2.09	191.39	0.425	25
